# Dr. Wm. A. Hawley

**Published:** 1891-07

**Authors:** 


### DR. WILLIAM A. HAWLEY.

William Agur Hawley, M. D., died at his home, No. 407 Montgomery Street, Syracuse, N. Y., Saturday morning, May 16th, after an illness of about three months. Heart difficulty developed a few years ago, and he became conscious then that his days were numbered. He was one of the oldest homœopathic practitioners in Central New York, and adhered rigidly and conscientiously to his convictions in regard to the practice of medicine.

Dr. Hawley was born August 28th, 1820, in Hinsdale, Berkshire County, Massachusetts. He was a son of Rev. W. A. Hawley, a Congregational minister, who preached for twenty-five years in that place, the Hawley family being descendants of Joseph Hawley, who settled in Stratford, Connecticut, about 1630. Dr. Hawley was fitted for college by his father, and when eighteen years old, entered Williams College, and graduated with credit in 1842. He first turned his attention to teaching, going for that purpose to Kentucky. In the winter of 1848 he returned to New England and took up the study of medicine, and graduated from the Albany (New York) Medical College in 1851, as he himself expressed it, "a confident believer in allopathy." He began the practice of medicine in Albany and in a few years turned his attention to Homœopathy.

From Albany he went to Saratoga Springs and associated himself with Dr. Bedortha in the "water cure" at that place. He then took charge of the "water cure" establishment at Lebanon Springs, Columbia County, which was the first "water cure" establishment in this country, and was very successful. After a year or so spent at Lebanon, he removed to Watertown, and in 1861 he came to Syracuse and associated himself with Dr. A. R. Morgan, and has practiced there ever since. He was one of the oldest homœopathic physicians in the country, and his practice was characterized by a strict obedience to its laws. He was a thinker, not alone on the practice of medicine, but on many subjects which claimed his attention. His position in his profession was in the front rank, and he was honored by his brethren in many ways, having held the office of President of the County Homœopathic Society eight years out of the twenty-seven of its existence. He was a member of the International Hahnemannian Association, of which he was President in 1888. He was also a member of the Central New York Homœopathic Society.

In September, 1851, he was married to Miss Willard, of Massachusetts, who died in 1889. He leaves three children, Mary E., William A., of Pittsburgh, Pa.; and Mrs. M. J. Howes, of Holyoke, Mass.

The Onondaga County Homœopathic Medical Society adopted these resolutions:

"WHEREAS, Death has entered our ranks, choosing therefrom a shining light in the person of Dr. William A. Hawley; be it

"*Resolved*, That through the death of Dr. Hawley this Society has lost one of its oldest, most valued, and respected members, one whose place it will be hard to fill; a man whose natural abilities would have rendered him prominent in any walk of life, whose professional attainments had gained him deserved eminence, whose stability and devotion to principle were most remarkable, and whose professional consecration was most thorough—a wise counselor and a leader of men.

"*Resolved*, That this Society pays highest tribute to Doctor Hawley's untiring efforts for the cause of Homœopathy, to his invaluable services in behalf of this organization, to his long years of effectual work among the sick, and to his value in the profession and to his fellow-men.

"*Resolved*, That we extend to his afflicted family our most cordial sympathy, together with expressions of our own personal bereavement.

"*Resolved*, That a copy of these resolutions be sent to the family of the deceased, to the *Medical Advance*, and that they be engrossed upon the records of the Society.

"J. W. SHELDON, M. D.,
"S. L. GOULD-LEGGETT, M. D.,
"K. ELMER KEELER, M. D.,
"*Committee.*"

